# Supplementary material for: Rapid Sequencing of Multiple RNA Viruses in Their Native Form
Source: Front Microbiol. 2019 Feb 25;10:260. doi: 10.3389/fmicb.2019.00260 (PMC6398364; doi:10.3389/fmicb.2019.00260)

Supplementary Figure S1: The details of mismatches and the general quality of the reads mapping for Figure 2 panel A and B are visualized as IGV snapshot. On the mapped reads lane, gray represents perfect match and other color represents mismatch.

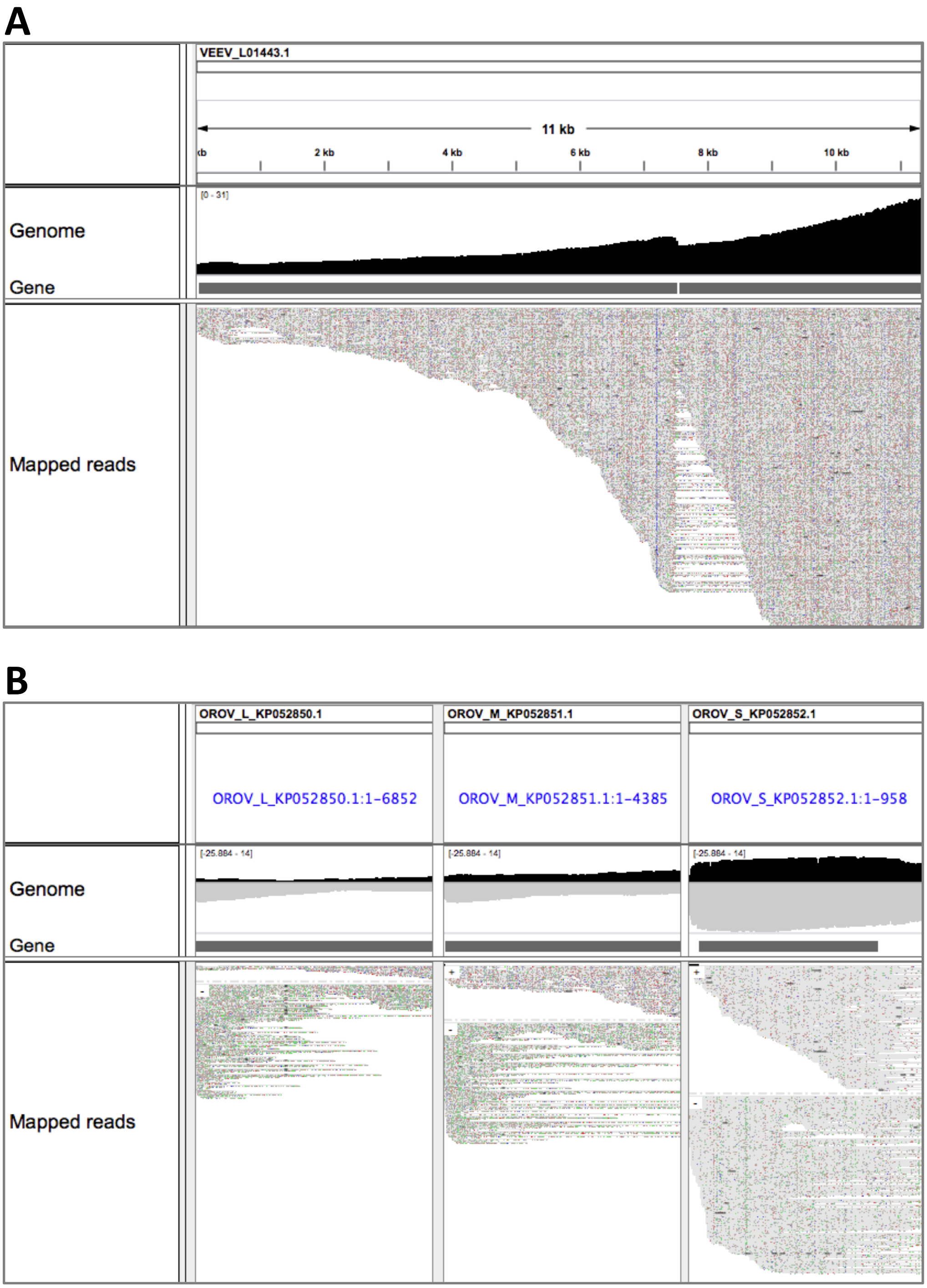

Supplement: Supplementary file 1 [file Data_Sheet_1.PDF]
